# Supplementary material for: Female students’ perspectives on cervical cancer screening inclusion in pre-admission university requirements in Ghana
Source: PLOS Glob Public Health. 2023 Jun 14;3(6):e0001029. doi: 10.1371/journal.pgph.0001029 (PMC10266602; doi:10.1371/journal.pgph.0001029)
Supplement: Attachment — Submitted filename: Response to Reviewers CCS.docx [file pgph.0001029.s001.docx]

| REVIEWER | COMMENT | RESPONSE |
| --- | --- | --- |
| REVIEWER 1 | Reviewer #1: Although the manuscript raises an interesting questions there are critical areas that calls for attention. Objective: "explore the views of female students towards inclusion of CCS to the preadmission screening" requirement. This is too generic an objective with potential interpretations. What does view refers to when in specific during permission etc could have been specified as specific objectives. | The study objective have been revised |
|  | Introduction: Given there are a lot of studies on CCS and probably dearth of evidence on students, the introduction section didn't provide convincing gaps that calls for this study. What other evidences are there in connection to students, admission etc is lacking from the introduction section. | Information regarding cervical cancer screening among female students have been included in the introduction as suggested see track changes |
|  | Method - While CCS has been well studied, and given exploratory-descriptive design is meant to explore for the unknown, the choice of the design should have been justified. | The reason for using exploratory descriptive design has been described as suggested |
|  | Study participants are selected purposively but which class year and whee they have come from was not specified. This made it difficult to understand if the principle of maximum variation is taken in to consideration. | The students from the selected university who were purposively recruited from all levels 100-400 |
|  | It is mentioned that "... choosing subjects who are believed to have fair knowledge about the content of the study and possess certain characteristics in which the researchers are interested in studying". What is fair knowledge and possession of certain characteristics should be clearer. | This statement has been revised and shown in track changes |
|  | Saturation - I am sure saturation may not happen at the same number for all questions. However, while explaining this may help, the last number at which saturation happens should be indicated.  It is stated that "The study instrument was first pretested on two females at a private Christian university in Ghana". I am wondering on why pretesting considered given there could be additional insights from the interview which may help sharpen the questions as you go along? any justification??. In fact you mentioned that "researchers penned down issues of interest in their diaries during the interview to aid in probing for further clarification" which is an indication of an ongoing calibration of tool during data collection | The sample size has been included  Thanks, the rationale for pretesting has been added  This has been clarified |
|  | Data analysis is poorly presented including the choice of content analysis as a frame. Content analysis is about counting contents which is not the case from the findings and secondly themes and meanings were mentioned which is not aligned with content analysis. You may need to read again on content analysis and if you wish to maintain this for your analysis. Check the word crowding in a soft ware of your finding to present the finding interns of which contents appeared more. In this section, you have a title about rigour which in reality is about ethics. You need to explain what was done to ensure quality/rigor. |  |
|  | Result - The result section requires major revision to tighten the different sections of the findings.  Critical is to have code book of themes, sub themes with explanations of what each stands for.  Besides, it is critical to read through this section to refine the arguments.  In fact there are shallow/crude words/statements in with such points as 'sexually active, both positive and negative views, and the theme themselves' that needs to be clarified and substantiated.  Please avoid some, more, few ... which doesn't make sense unless you contextualise in the form of -- out ---.  There are repetitions of findings that could be pulled under support, refute.... good examples are hesitation, reluctance, fear... which could be part of refuting. Please revise the themes to be retitled.  There are fallacies where willingness for screening was presented. While the students are already on campus their response on willingness for screening doesn't make sense. So, if the focuse is about pre-admission remain focused on that or if you want to measure both pre-admission and those already on campus then rework on your objective.... | Thanks, the results section has been revised extensively as suggested  The themes and subthemes have been revised  The table 2 has the themes and subthemes generated  This section has been revised as recommended  Words like sexually active, negative and positive have been replaced  The terms have been clarified  Reluctance and hesitance have been used to replace some of the words to avoid redundancy  Willing for screening was an emerging theme based on their responses |
|  | Discussion - Give there are some irregularities, crude statements, repetitions in the result section, the discussion section is not tight. What is it that this study could be known of? From this section it gives an impression that support and willingness themes are VERY IMPORTANT findings that is chosen for interpretation. Nonetheless, what was discussed about these remains too shallow and unwarranted. What is the outstanding finding from this study from the different theme or just one theme. How do you interpret this - what does this mean in view of available evidences? I think this part is too shallow. | The discussion section has been revised as suggested |
|  |  |  |
|  |  |  |
