# Supplementary material for: Female students’ perspectives on cervical cancer screening inclusion in pre-admission university requirements in Ghana
Source: PLOS Glob Public Health. 2023 Jun 14;3(6):e0001029. doi: 10.1371/journal.pgph.0001029 (PMC10266602; doi:10.1371/journal.pgph.0001029)
Supplement: Attachment — Submitted filename: RESPONSE TO COMMENTS.docx [file pgph.0001029.s002.docx]

| **REVIEWER** | **COMMENTS** | **RESPONSE** |
| --- | --- | --- |
| REVIEWER 2 | Needs to be more organized with details about cervical cancer screening guidelines followed in Ghana (Age recommendation for screening, whether done free of cost/paid, mandatory/optional) to make it more understandable for international readers.  Clarify whether the recommendation for inclusion of CCS in pre-admission screening requirement was made by Government in Ghana or this study is based on recommendation made by authors of previous studies. | Thank you for taking the time to review this manuscript and for the comments made to enhance the study  There are no national policy guidelines for CCS in Ghana and hence is based on recommendations by institutions such as ACS, WHO, etc This has been noted in the study  Cost of information has been included.  These were recommendations made by the study participants (The students themselves) |
| REVIEWER 2 | Mention the gender, qualification and training in qualitative research of the person who collected the data  Provide details about the questions included in the semi-structured questionnaire | The information requested have been provided  Details about the questionnaire have been provided as suggested and highlighted in the method section |
| REVIEWER 2 | Data analysis Mention how many authors were involved in the coding process and their training in qualitative research methods The terms “categories and sub-categories” and “Themes and sub-themes” are used interchangeably by the authors in the data analysis and results section | Information has been provided as requested  This has been resolved and highlighted thank you |
| REVIEWER 2 | Results: While presenting socio-demographic characteristics of the participants, use either words or number to present the no. of participants in each category. Ex: Eighteen (18)-avoid using both.  In Table 1: Clarify what is this variable “Level” represents.  The results presented in Table 2 does not match with the Content analysis framework mentioned by the authors to have been used for analysis | One of them has been maintained as suggested  Students academic level  Clarifications have been made for the table and the content analysis section to correspond as suggested. |
| REVIEWER 2 | Conclusion: Conclusion provided by the authors “Undoubtedly, CCS has been proven effective in helping to reduce the incidence rate and the burden of cervical cancer” was not based on the findings of the present study | The conclusion section have been revised to include the main findings |
| REVIEWER 2 | References: There are errors in citing the references (Ex: 4,20,29). Check how to cite references for online resources, books etc | Reference 4, 20 and 29 have been revised as suggested. |
